# Low-cost three-dimensional printed phantom for neuraxial anesthesia training: Development and comparison to a commercial model

**DOI:** 10.1371/journal.pone.0191664

**Published:** 2018-06-18

**Authors:** Azad Mashari, Mario Montealegre-Gallegos, Jelliffe Jeganathan, Lu Yeh, Joshua Qua Hiansen, Massimiliano Meineri, Feroze Mahmood, Robina Matyal

**Affiliations:** 1 Department of Anesthesia and Pain Management, Toronto General Hospital, Toronto, Ontario, Canada; 2 Faculty of Medicine, University of Toronto, Toronto, Ontario, Canada; 3 Department of Anesthesia, Critical Care and Pain Medicine, Beth Israel Deaconess Medical Center, Harvard Medical School, Boston, Massachusetts, United States of America; 4 Department of Anesthesiology, University of Groningen, University Medical Center Groningen, Groningen, The Netherlands; Georgetown University Medical Center, UNITED STATES

## Abstract

Neuraxial anesthesia (spinal and epidural anesthesia) procedures have significant learning curves and have been traditionally taught at the bed side, exposing patients to the increased risk associated with procedures done by novices. Simulation based medical education allows trainees to repeatedly practice and hone their skills prior to patient interaction. Wide-spread adoption of simulation-based medical education for procedural teaching has been slow due to the expense and limited variety of commercially available phantoms. Free/Libre/open-source (FLOS) software and desktop 3D printing technologies has enabled the fabrication of low-cost, patient-specific medical phantoms. However, few studies have evaluated the performance of these devices compared to commercially available phantoms. This paper describes the fabrication of a low-cost 3D printed neuraxial phantom based on computed tomorography (CT) scan data, and expert validation data comparing this phantom to a commercially available model.

**Methods:**

Anonymized CT DICOM data was segmented to create a 3D model of the lumbar spine. The 3D model was modified, placed inside a digitally designed housing unit and fabricated on a desktop 3D printer using polylactic acid (PLA) filament. The model was filled with an echogenic solution of gelatin with psyllium fiber. Twenty-two staff anesthesiologists performed a spinal and epidural on the 3D printed simulator and a commercially available Simulab phantom. Participants evaluated the tactile and ultrasound imaging fidelity of both phantoms via Likert-scale questionnaire.

**Results:**

The 3D printed neuraxial phantom cost $13 to print and required 25 hours of non-supervised printing and 2 hours of assembly time. The 3D printed phantom was found to be less realistic to surface palpation than the Simulab phantom due to fragility of the silicone but had significantly better fidelity for loss of resistance, dural puncture and ultrasound imaging than the Simulab phantom.

**Conclusion:**

Low-cost neuraxial phantoms with fidelity comparable to commercial models can be produced using CT data and low-cost infrastructure consisting of FLOS software and desktop 3D printers.

## Introduction

Procedural training during anesthesiology residency has traditionally occurred as a byproduct of patient care where learning is not the primary objective of the procedure[1–3]. Recently implemented restrictions in resident duty hours, and the transformation of training from a time-based apprenticeship to a competency-based model, have highlighted a need for better and more efficient training techniques. Simulation has the potential to improve procedural training by allowing trainees to acquire proficiency in a realistic environment where the only consequence of error is learning[4,5]. This allows trainees to become “experienced novices” before clinical procedural practice. While procedural simulations have improved significantly over the past decade, the greatest success has been achieved for procedures that involve video displays and mechanical operator interfaces, such as laparoscopic surgery, endoscopy, and echocardiography[6–11].

Neuraxial anesthesia is based largely on tactile feedback and is increasingly supplemented by pre-procedure ultrasound imaging. While commercial simulators and phantoms for neuraxial anesthesia training are commercially available, widespread use and incorporation into residency curricula has been limited by several factors including significant costs. Most commercial neuraxial simulators have unit costs in the thousands of dollars. While the phantoms typically last several years, the cost of the models and the resulting small numbers that can be purchased by training programs limit access for most trainees, who are often distributed across multiple teaching sites. In addition the high cost limits the variety of models that are available and the ability to simulate normal and pathologic variants. The development of sophisticated free/libre and open-source (FLOS) software for the processing of medical imaging data and high quality, low-cost three-dimensional (3D) printing have allowed for the fabrication of high-fidelity models derived from patient imaging data at significantly lower costs. Similar models have been produced using commercial software and industrial-grade 3D printers to create training phantoms for neuraxial anesthesia[12]. More recent studies have shown that this process can be used to fabricate patient-specific neuraxial simulators using free/open-sourced software and desktop 3D printing technologies[13,14]. However, there is currently no data comparing the performance of low-cost desktop 3D printed phantoms with existing commercial systems. Therefore, our main objective was to develop a low-cost neuraxial anesthesia phantom and compare its fidelity to that of a commercially available model.

## Methods

Our hospital’s Institutional Review Board approved this study as part of a project for improvement of ultrasound education. Faculty members from the Department of Anesthesia, Critical Care and Pain Medicine were invited to participate in the study. Informed consent was implied if the faculty member decided to participate.

## 1. Creation of the neuraxial anesthesia phantom model

**Fig 1:**
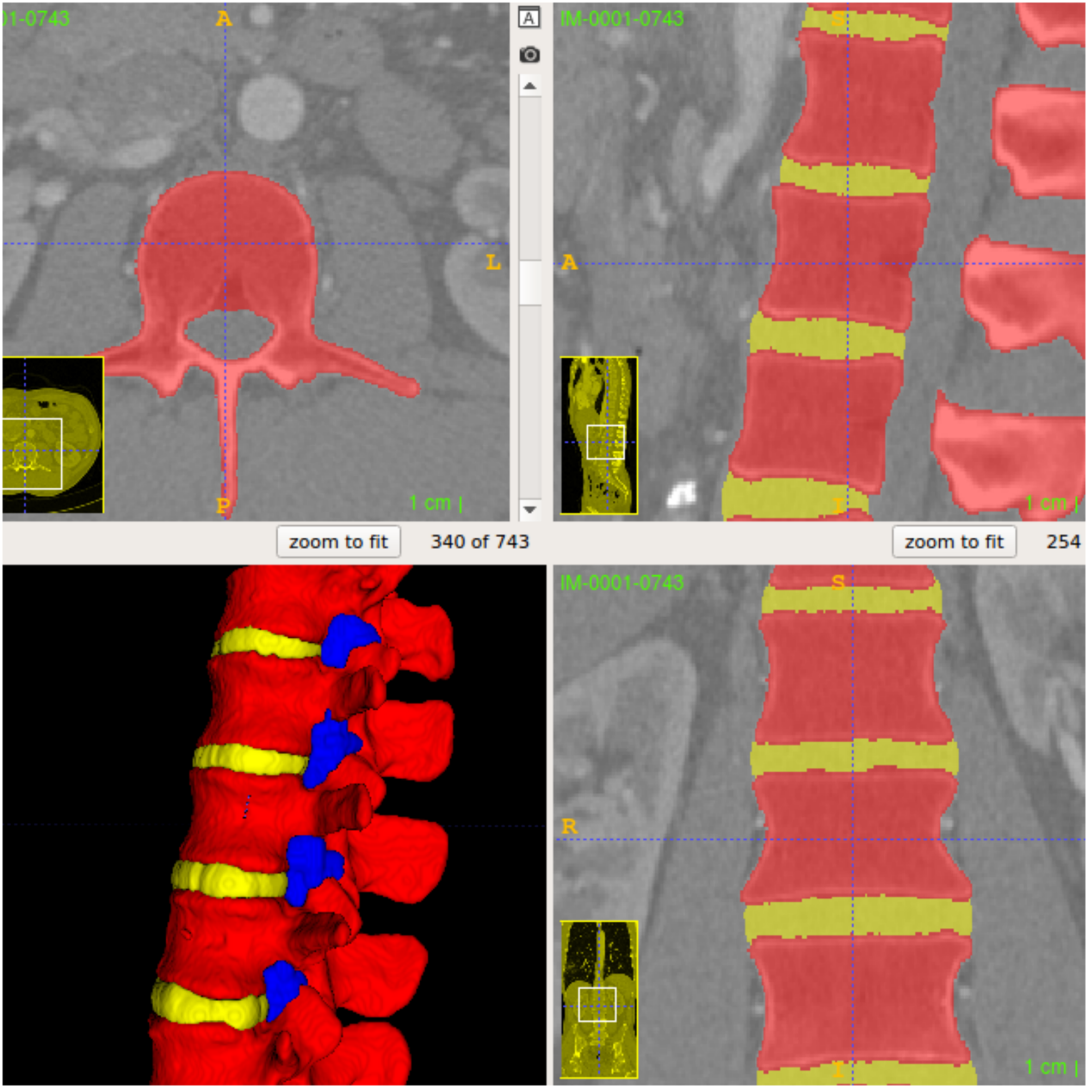
ITK-SNAP interface after segmentation of the spine with the automated active contour tool. Each colour represents a different layer-mask indicating different regions for the segmented 3D model.

**Fig 2:**
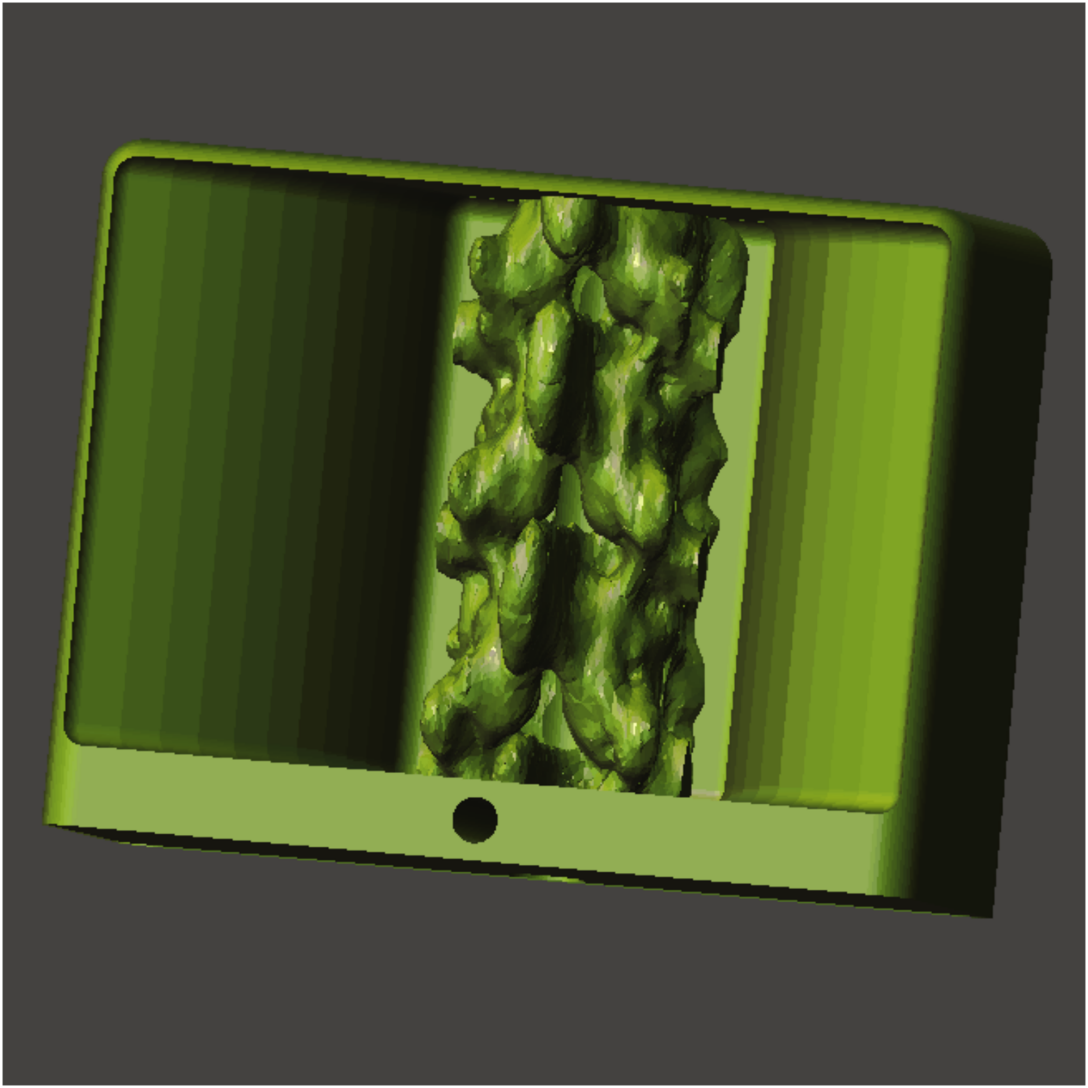
Final 3D model of the neuraxial simulator before 3D printing. The segmented spine model is combined with a CAD-designed enclosure. This enclosure provides a receptacle to hold the echogenic gelatin medium.

An anonymized computerized tomography (CT) data set of the human torso was obtained as a Digital Communication in Medicine (DICOM) file from the website www.osirix-viewer.com/datasets. At the time of this study, the DICOM was available freely for research and educational use. The dataset was free of any spinal pathology. We used the FLOS software ITK-SNAP (http://www.itksnap.org) and 3DSlicer for image segmentation[15]. The process was performed using semi-automated active contour (“snake”) tool. In-depth explanation of the active contour tool can be found in the ITK-SNAP online tutorials[16] (Fig 1). The accuracy of the segmentation was verified by one of the authors (AM), a trained anesthesiologist experienced with spinal anatomy and imaging. Following the segmentation, the models were converted and exported as surface mesh files in stereolithography (.stl) format. The 3D model was imported into FLOS computer assisted design software OpenSCAD (http://www.openscad.org/). The spine model was cropped and merged with a box enclosure to create a single object (Fig 2). The box enclosure was designed to hold gelatin or other echogenic matrix material and allow mounting of the final phantom to a desk using a clamp.

The model was imported into FLOS slicing software Cura version 15.02.1 (Ultimaker, Geldermalsen, Netherlands) with printer configurations customized for a FLOS hardware fused deposition modeling (FDM) 3D printer with a 0.5 mm Wade’s extruder (Lulzbot Taz 5, Aleph Objects, Loveland, CO) equipped with polylactic acid (PLA) printing filament (eSUN, Shenzhen Esun Industrial Co., Shenzhen, China). Print settings for PLA are summarized in Table 1. Once parameters were set, each spine model was exported (as a.gcode file) and uploaded to the printer.

**Table 1:** Slicing settings.

**Perimeter thickness (mm)**	1.5
**Infill (%)**	20
**layer height (mm)**	0.2
**Extruder temperature (°C)**	205
**Bed temperature (°C)**	60

**Fig 3:**
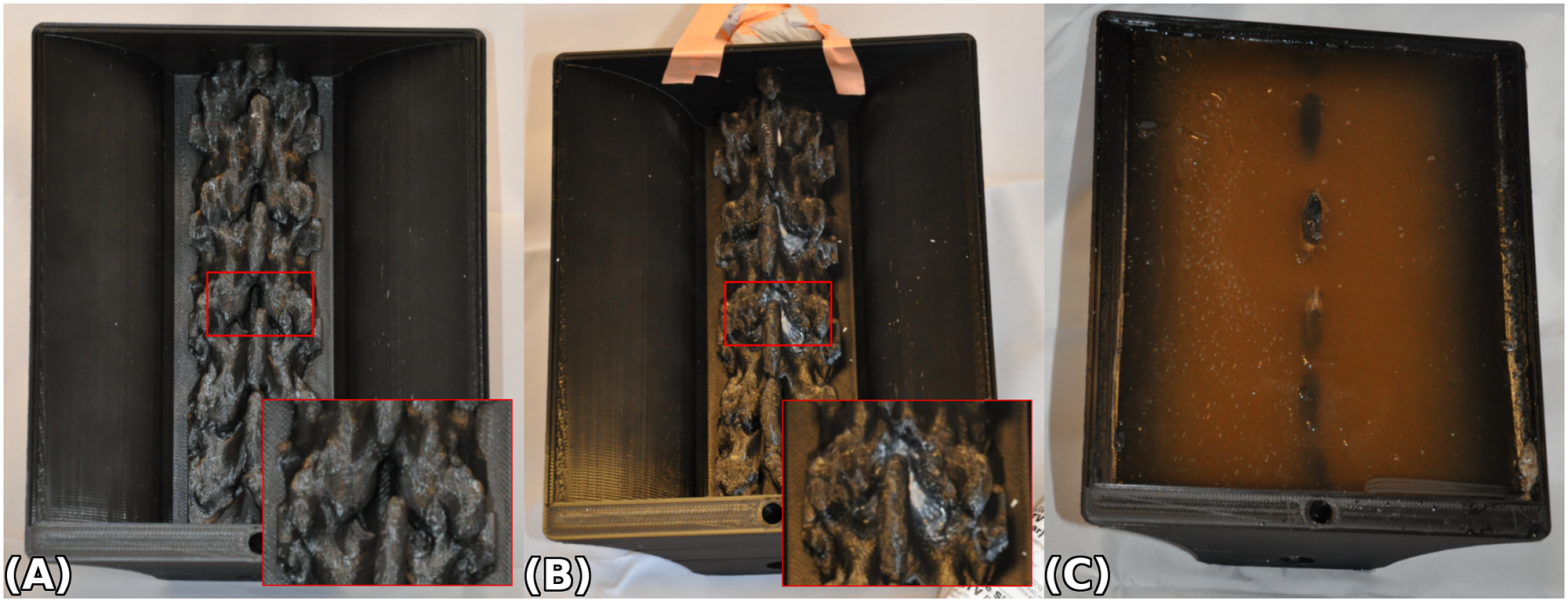
Process to create echogenic components of the 3D printed epidural simulator. (A) 3D printed neuraxial phantom with inset showing enlarged view of one interspace. (B) Modeling the ligamentum flavum with silicone paste with inset showing enlarged view of a single level. (C) Total submersion of spine with echogenic gelatin solution (9.0% w/v).

After the model was printed (Fig 3A), the ligamentum flavum was manually modeled over the spine model using a silicone paste (Silite Silicone RTV Sealant, ITW Devcon, Danvers, MA) and allowed to set for 2 hours (Fig 3B). The finger of an inflated examination glove was used to prevent the silicone paste from filling the spinal canal. A 9.0% w/v gelatin solution was made by mixing 90 g of un-flavored Knox gelatin (Kraft Foods Group Inc., Northfield, IL) with 1L of cold tap water. The mixture was left to rest for 30 minutes and then heated until the gelatin was fully dissolved[17]. The solution was refrigerated for 3 hours until set, and then heated again to melting point. The cycle was repeated 2 times in order to increase the stiffness of the gelatin. During the last cycle, 4 g of psyllium fiber (Metamucil Brand, Proctor & Gamble, Connecticut, OH) was added as a scattering agent, and mixed until fully dissolved[18]. After the solution cooled to approximately 30°C it was poured into the model enclosure fully covering the spine. The model was refrigerated overnight (9–10°C) (Fig 3C).

A silicone (Mold star 15, Smooth-on, Macungie, PA) cylinder with one sealed end was cast from a 3D printed mold to simulate the thecal sac. The cylinder was inserted into the spinal canal of the phantom with the open end at the top and connected to a 1 L bag of normal saline solution using a standard intravenous infusion line.

Data files for the phantom are available for download at https://github.com/tgh-apil/LumbarSpinePhantom.

## 2. Comparison to a commercial model

The 3D printed neuraxial phantom was assessed against the Simulab Lumbar Puncture/Epidural Ultrasound-guided Procedural Trainer (Simulab Corporation, Seattle, WA). This system offers one normal and three pathological lumbar spine modules (geriatric, obese and obese geriatric). For this study we used the normal model.

We invited twenty-two staff anesthesiologists from one of our hospitals to use and evaluate the 3D printed neuraxial phantom and the Simulab Procedural Trainer. Participants were asked to perform one spinal and one epidural procedure on each phantom. The order of the phantoms was alternated across participants. Participants that reported routine use of ultrasound for neuraxial procedures were asked to perform an ultrasound assessment of the models in transverse, median sagittal and paramedian sagittal views, using a standard ultrasound system (GE Logiq E, GE Healthcare, Chicago, IL). The participants were asked to rate the tactile and ultrasound fidelity of the models using an anonymous Likert-scale questionnaire (S1 File).

## 3. Data analysis

Data was transcribed into a spreadsheet and analyzed using LibreOffice Calc Version 5.1.3 (The Document Foundation, Wiesbaden, Germany). Data are reported as median [interquartile range]. Mann-Whitney paired-sample t-tests were used for comparison tests. While the ordinal data in this study does not strictly fulfill the underlying assumptions of parametric tests, the robustness of such tests and their appropriateness for use in this context has been well established[19–21].

## Results

In total, the 3D printed neuraxial phantom required 423 g of PLA filament to print at a cost $13. Twenty-five hours of non-supervised 3D printing time were used. The segmentation of the spine from C, the creation of the 3D model, assembly and gelatin preparation (not including time for gelatin to set) required an additional 6–8 hours.

Twenty-one of 22 participants performed spinal and epidural needle placement on both models (one participant only submitted ratings for one simulator; these ratings were discarded). Sixteen participants reported routine use of ultrasound for neuraxial anesthesia and submitted ratings for the ultrasound images. The median experience as attending anesthesiologist was 11 years, with a range of 0–34 years. Thirteen participants (59%) reported performing more than 2 spinals per month. The same number reported performing more than 2 lumbar epidurals per month. Three and 5 participants reported performing more than 10 spinals and epidurals per month, respectively. Ratings were on a 1 to 5 scale with 1 indicating “Poor”, 2 “Fair”, 3 “Average”, 4 “Good”, and 5 “Excellent” fidelity to clinical reality.

There was one case of procedural failure for spinal in which the participant was unable to get cerebrospinal fluid return from the Simulab phantom. During epidural placement, there were 9 (41%) unintended dural punctures on the Simulab phantom, and 1 (5%) with the 3D printed model. Likert-scale rating results are summarized in Table 2.

**Table 2:** Expert ratings of the 3D-printed (3DP) and Simulab phantoms. Ratings where on a 1 to 5 scale with 1 = Poor, 2 = Fair, 3 = Average, 4 = Good and 5 = Excellent fidelity to clinical reality.

	Simulab	3DP Phantom	
	Median [IQR] Mean/SD/Range	Median [IQR] Mean/SD/Range	*p*
**Tactile Fidelity**			
Surface palpation	**4 [3–5]** 4.0/0.9/2-5	3 [2–4] 3.1/1.4/1-5	**0.003**
Soft tissue resistance	3 [3–4] 3.5/1.1/1-5	3.5 [3–4] 3.4/0.9/1-5	0.847
Lig. flavum resistance	3 [2–4] 2.9/1.1/1-5	**4 [3.25–4.75]** 4.0/0.9/2-5	**0.016**
Loss of resistance (epidural)	2 [2–4] 2.8/1.4/1-5	**4 [4–5]** 4.2/0.8/2-5	**0.009**
Dural puncture (spinal)	3 [2–4] 3.0/1.3/1-5	**4 [3–4]** 3.9/0.9/2-5	**0.008**
**Ultrasound Imaging Fidelity**			
Transverse view	3 [2.75–4] 2.9/1.0/1-5	**4 [3–5]** 4.3/0.8/2-5	**0.037**
Median sagittal view	3 [2–4] 2.7/1.1/1-5	**4 [3.75–5]** 4.2/0.7/2-5	**0.014**
Paramedian sagittal view	3 [2–4] 2.6/1.2/1-5	**4 [3.75–4.25]** 4.1/0.7/2-5	**0.014**
**Appropriateness for Educational Use**			
Novice trainees			
- Spinal anesthesia	3 [2–4] 3.1/1.2/1-5	**4 [3–4]** 3.8/0.7/3-5	**0.003**
- Epidural anesthesia	3 [3–4] 3.2/1.3/1-5	**4 [3–5]** 4.1/0.8/3-5	**0.016**
Refresher for trained staff			
- Spinal anesthesia	3 [1.5–3.5] 2.6/1.2/1-4	3 [2–4] 3.2/1.1/1-5	0.3
- Epidural anesthesia	3 [1.5–3.5] 2.6/1.2/1-4	3 [2–4] 3.2/1.1/1-5	0.3

### Tactile fidelity

**Fig 4:**
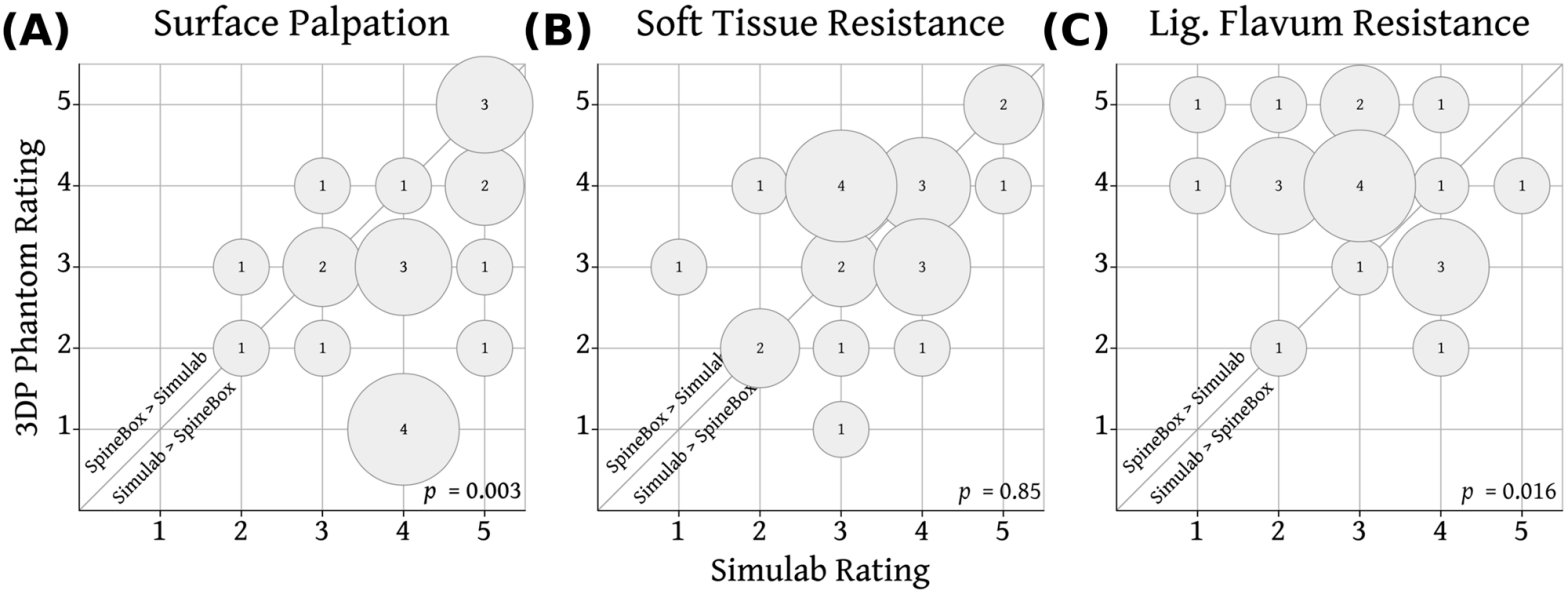
Comparison of tactile fidelity for surface palpation, soft tissue resistance and ligamentum flavum resistance between Simulab and the 3DP Phantom. Rating for the 3D Phantom from each expert is plotted against the expert’s rating of the Simulab Phantom. Size of bubble and number indicate the number of experts. Bubbles above the diagonal line represent experts rating the 3D Phantom higher than the Simulab Phantom.

**Fig 5:**
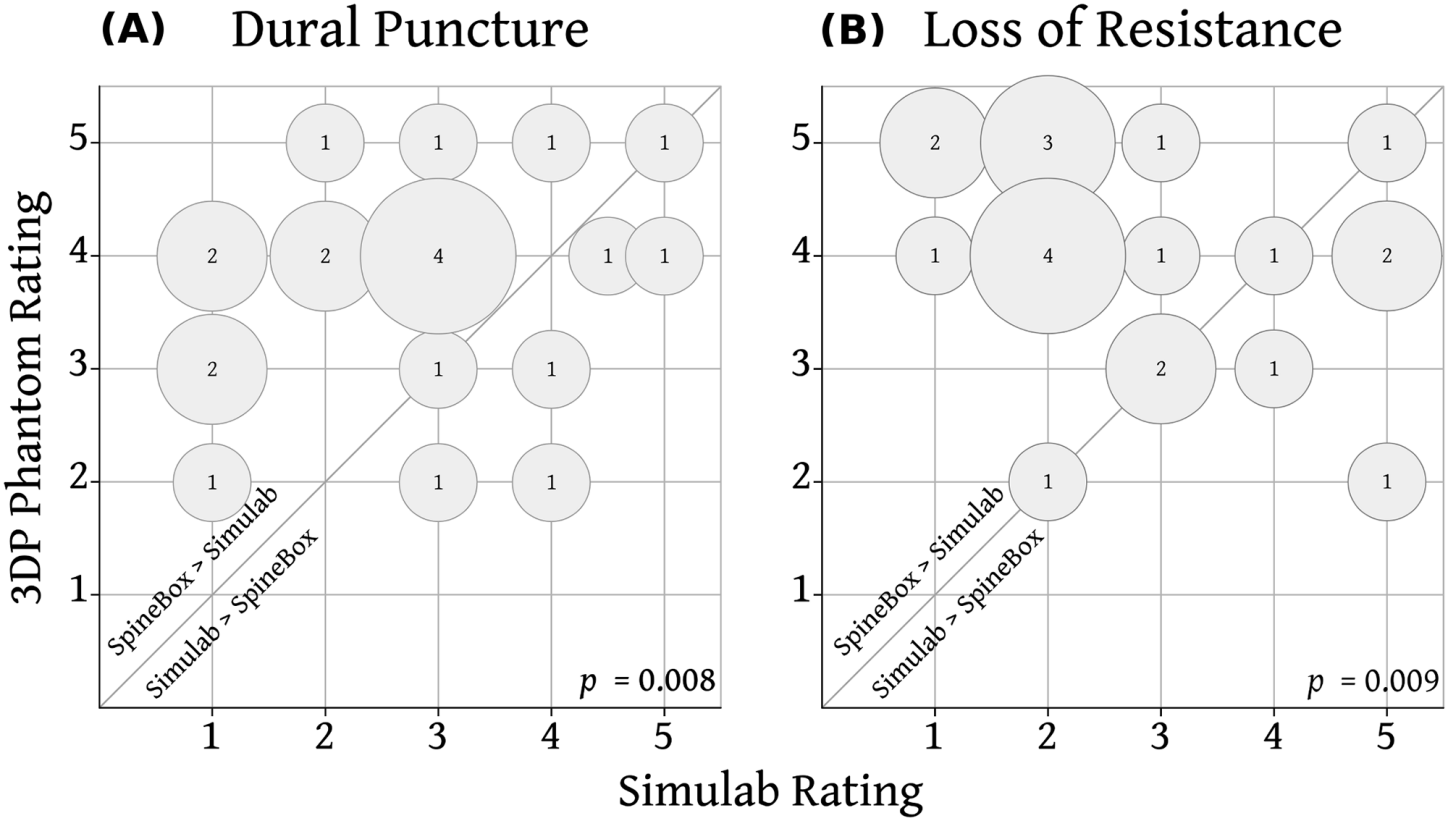
Comparison of tactile fidelity for loss of resistance (epidural) and dural puncture (spinal) between Simulab and the 3DP Phantom. Rating for the 3D Phantom from each expert is plotted against the expert’s rating of the Simulab Phantom. Size of bubble and number indicate the number of experts. Bubbles above the diagonal line represent experts rating the 3D Phantom higher than the Simulab Phantom.

Paired expert ratings for tactile fidelity of the two phantoms are shown in Figs 4 and 5. Fidelity for surface palpation was rated significantly higher for the Simulab phantom (Fig 4A). The gelatin in the 3D printed phantom was found to be too soft and fragile for palpation by most (12 of 22) experts. There was no significant difference between the two phantoms in the fidelity of soft-tissue resistance to needle (Fig 4B). Ligamentum flavum resistance (Fig 4C), dural puncture (spinal) (Fig 5A) and loss of resistance (epidural) fidelity (Fig 5B) were all rated significantly higher for the 3D printed phantom.

### Imaging fidelity

**Fig 6:**
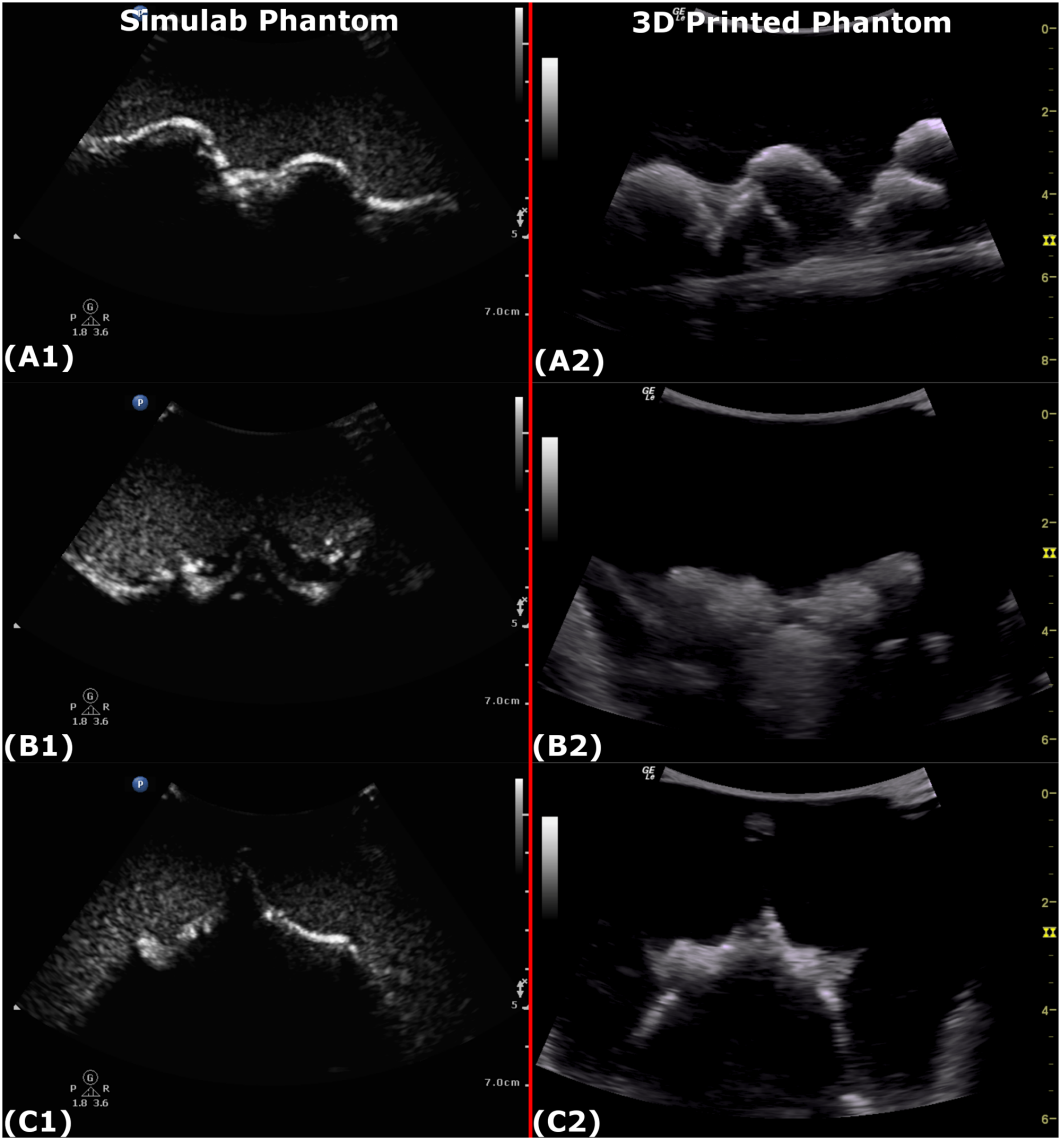
Ultrasound images of the Simulab and 3D printed phantoms. Ultrasound views were taken with a linear ultrasound probe. Images from the Simulab phantom are shown in the left column, those from the 3D printed phantom in the right. Images collected were: (A1, A2) Paramedian sagittal view, (B1, B2) Transverse view at inter-space and (D1, D2) Transverse view at spinous process.

**Fig 7:**
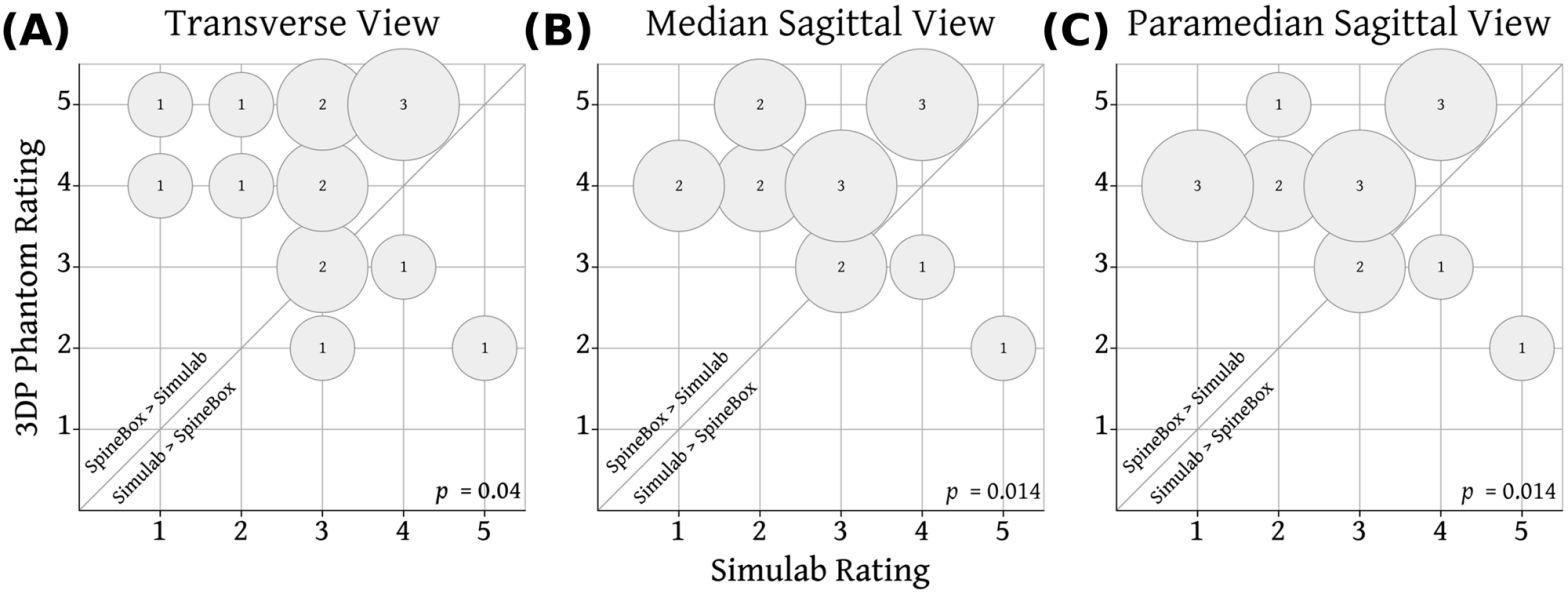
Comparison of ultrasound imaging fidelity for Simulab and the 3DP Phantom. Rating for the 3D Phantom from each expert is plotted against the expert’s rating of the Simulab Phantom. Size of bubble and number indicate the number of experts. Bubbles above the diagonal line represent experts rating the 3D Phantom higher than the Simulab Phantom.

Ultrasound images of the 3D printed neuraxial phantom are displayed in Fig 6. Overall, the PLA and gelatin effectively mimicked the echogenicity of bone and soft-tissue respectively. Paired ratings of ultrasound imaging fidelity from the two phantoms are shown in Fig 7. There was little variation in the ratings across different views. Overall the images from the 3D printed phantom were preferred by the majority (11–12 depending on the view); rated as equivalent by 2 and inferior to the Simulab images by 2–3 of the 16 experts.

### Appropriateness for educational applications

The 3D printed phantom had a median rating of 4 (“Good”) for appropriateness for novice training in both spinal and epidural anesthesia. The Simulab phantom has a median rating of 3 (“average”) for both applications. Both phantoms had a median rating of 3 (“average”) for use as a refresher tool for intermediate or fully trained practitioners (Table 2).

## Discussion

We were able to develop a high-fidelity neuraxial anesthesia phantom using FLOS software, a desktop 3D printer, and low-cost materials. Both phantoms were used and evaluated by experts and the 3D printed neuraxial phantom was found to be comparable to the commercial Simulab model. Such 3D printed phantoms can be deployed in residency programs, including in resource-constrained settings, to facilitate procedural training for neuraxial anesthesia. Variations of this model, including pathologies, could be easily generated from other patients’ CT data and digitally manipulated to create a wide variety of patient-specific phantoms with varying levels and types of difficulty.

Most 3D printed models reported to date use expensive infrastructure in the form of industrial printers and proprietary image processing software which require specialized expertise to maintain and operate. While the printing services can be outsourced, the image processing software required in-house for the generation of the models has annual licensing costs in excess of $10000. This expensive investment into infrastructure development limits the availability of these technologies to well-funded medical centers thereby limiting the widespread use of these simulators across training programs. Our method results in a significant reduction of both the capital (3D printer with a one-time cost of < $3000, FLOS software) and unit costs (< $10–40) of production.

While the unit cost of the 3D printed phantom is far lower than that of commercial models, this cost saving has to be balanced against the time, infrastructure and expertise required to create such phantoms. The increasing quality and availability of accessible software and printing infrastructure, increasing computational literacy amongst clinicians and medical educators, and platforms for sharing 3D models are making the large scale deployment of customized, patient-specific training phantoms such as the one presented here ever more realistic. But for situations where one or a few phantoms will suffice and where financial resources are more readily available, commercial phantoms can be more conveniently and rapidly obtained.

Our study has some potential limitations. First, due to the clear differences in the models’ appearance the participants could not be blinded. Second, as an initial evaluation, we tested the models only in faculty members and not trainees. The experienced participants were able to rate the fidelity of the phantoms to real-life clinical situations. Trainees with limited clinical experience could not have reliably provided such information. However further studies with trainees are required to ascertain the educational efficacy of this approach compared to traditional teaching methods as well as other methods based on patient-specific 3D models, such as virtual reality simulations. While physical phantoms provide haptic feedback, allow the use of standard instruments and adjunctive techniques such as ultrasound guidance, virtual reality simulations can be easier to implement and more flexible to deploy when the necessary infrastructure is available and can potentially be more immersive.

There was an unexpectedly high incidence of dural puncture in the commercial phantom. The incidence of dural puncture during lumbar epidural procedures has been frequently reported at between 1–4% in teaching centers [22–24], far below the 41% incidence in the Simulab phantom in the hands of expert practitioners. Due to lack of access to the internal components of the Simulab phantom we were not able to ascertain the cause of this. While the single dural puncture in the 3D phantom (5% incidence) is within range quoted in literature, our small sample does not allow us to rule out the possibility that the 3D printed phantom made the procedure too easy. However none of the participants mentioned this to be the case.

The 3D printed phantom also had three major limitations. First, there was a lack of realism in surface palpitation due to the softness of the gelatin solution used. Second, the perishable nature of the gelatin and psyllium media means that a new batch of gelatin would need to be created repeatedly—a time consuming process. Third, needle track marks persisted in the models after use. These issues will likely be addressed by the substitution of a ballistic gel (Clear Ballistics, Fort Smith, AR, USA) in place of gelatin. Ballistic gel is an inorganic substance, is non-perishable, may be re-used numerous times. Previous studies have demonstrated the echogenic properties of ballistic gel for imaging phantoms [25,26]. However, due to its higher melting point, the phantom will need to be printed from more heat tolerant materials, such as nylon. Doing so will only marginally increase the raw materials cost.

The materials used in the construction of the 3D printed phantom were chosen in an attempt to replicate the two essential contrasts in tissue resistance during epidural insertion: from soft-tissue (muscle and fat) to the more stiff ligamentum flavum, followed by a loss of resistance on entry into the epidural space. In real life there are often more subtle but potentially palpable transitions in the soft tissue layers encountered before the ligament. It is not currently feasible to replicate these without significant increase in cost and production complexity and to our knowledge this level of tissue fidelity is not captured by any commercial phantom currently available. We hypothesized that this limitation would not significantly affect the educationally relevant components of the model’s fidelity. This was largely supported by the expert ratings of the phantoms in this study.

## Conclusion

Low-cost 3D printed neuraxial anesthesia phantoms derived from patient CT data can be created with modest infrastructure and achieve fidelity comparable to commercial models. Such models allow increased utilization of phantoms for procedural training and can be customized for specific teaching applications and levels of difficulty.

## Supporting information

S1 File Spine phantom trial data collection form. Data collection form.
(PDF)
