# Supplementary material for: Low-cost three-dimensional printed phantom for neuraxial anesthesia training: Development and comparison to a commercial model
Source: PLoS One. 2018 Jun 18;13(6):e0191664. doi: 10.1371/journal.pone.0191664 (PMC6005480; doi:10.1371/journal.pone.0191664)
Supplement: S1 File — Data collection form. (PDF) [file pone.0191664.s001.pdf]

Spine Phantom Trial - Data Collection Form - 2016-02-19

Demographics

1 Years of full-time experience as staff/attending anesthesiologist: _______

2 On average how many **spinals per month** have you performed during the past year (including diagnostic and anesthetic)? (Circle): <2 2-5 5-10 >10

3 On average how many **epidurals** per month have you performed during the past year? (Circle)

3.1 Lumbar: <2 2-5 5-10 >10

3.2 Thoracic: <2 2-5 5-10 >10

Model Evaluation

1 Please rate the **tactile realism** of the following features
(1= Poor, 2= Fair, 3= Average, 4= Good, 5 = Excellent)

	Simulab Phantom	Box Phantom
1.1 Surface palpation	1 . 2 . 3 . 4 . 5	1 . 2 . 3 . 4 . 5
1.2 Needle resistance in soft tissue	1 . 2 . 3 . 4 . 5	1 . 2 . 3 . 4 . 5
1.3 Needle resistance in ligamentum flavum	1 . 2 . 3 . 4 . 5	1 . 2 . 3 . 4 . 5
1.4 Loss of resistance (epidural)	1 . 2 . 3 . 4 . 5	1 . 2 . 3 . 4 . 5
1.5 Dural puncture (spinal only)	1 . 2 . 3 . 4 . 5	1 . 2 . 3 . 4 . 5

Comments:

2 Please rate the realism of the **ultrasound views** (live scanning)

	Simulab Phantom	Box Phantom
2.1 Transverse (Bat wings) view	1 . 2 . 3 . 4 . 5	1 . 2 . 3 . 4 . 5
2.2 Median sagittal view	1 . 2 . 3 . 4 . 5	1 . 2 . 3 . 4 . 5
2.3 Paramedian sagittal view	1 . 2 . 3 . 4 . 5	1 . 2 . 3 . 4 . 5

Comments:

3 How useful would this model be for teaching or practice for following groups?

	Simulab Phantom	Box Phantom
**3.1 Novice trainee**		
3.1.1 spinal?	1 . 2 . 3 . 4 . 5	1 . 2 . 3 . 4 . 5
3.1.2 epidural?	1 . 2 . 3 . 4 . 5	1 . 2 . 3 . 4 . 5
3.2 **Intermediate trainee** (~CA-2)		
3.2.1 spinal?	1 . 2 . 3 . 4 . 5	1 . 2 . 3 . 4 . 5
3.2.2 epidural?	1 . 2 . 3 . 4 . 5	1 . 2 . 3 . 4 . 5
3.3 Refresher for **experienced trainees or practitioners** who have not performed the procedure for some time		
3.3.1 spinal?	1 . 2 . 3 . 4 . 5	1 . 2 . 3 . 4 . 5
3.3.2 epidural?	1 . 2 . 3 . 4 . 5	1 . 2 . 3 . 4 . 5

Comments:
